# Assessment of Frailty in Community-Dwelling Older Adults Using Smartphone-Based Digital Lifelogging: A Multi-Center, Prospective Observational Study

**DOI:** 10.3390/s26010215

**Published:** 2025-12-29

**Authors:** Janghyeon Kim, Namki Hong, Hee-Won Jung, Seungjin Baek, Sang Wouk Cho, Jungheui Kim, Changseok Lee, Subeom Lee, Bo-Young Youn

**Affiliations:** 1 Department of Style Tech, Hwasung Medi-Science University, Hwaseong-si 18274, Republic of Korea; jamie8122@naver.com; 2 Department of Internal Medicine, Endocrine Research Institute, Severance Hospital, College of Medicine, Yonsei University, Seoul 03722, Republic of Korea; nkhong84@yuhs.ac.kr (N.H.); 2022314257@o365.yonsei.ac.kr (S.B.); swcho20@yuhs.ac (S.W.C.); 3 Division of Geriatrics, Department of Internal Medicine, Asan Medical Center, College of Medicine, University of Ulsan, Seoul 05505, Republic of Korea; dr.ecsta@gmail.com; 4 Institute for Innovation in Digital Healthcare, Yonsei University Health System, Seoul 03722, Republic of Korea; bigdaheta@yuhs.ac; 5 Department of Biomedical Systems Informatics, College of Medicine, Yonsei University, Seoul 03722, Republic of Korea; 6 DYPHI Research Institute, DYPHI Inc., Songpa-gu, Seoul 05836, Republic of Korea; ckdckd145@dyphi.com (C.L.); sblee@dyphi.com (S.L.); 7 Department of Healthcare Management, College of Health and Medical Science, Daejeon University, Daejeon 34520, Republic of Korea

**Keywords:** frailty, older adults, mobile health, digital health, digital lifelogs

## Abstract

**Highlights:**

**What are the main findings?**

- Smartphone-based digital lifelogs, specifically usual gait speed, daily step count, and subjective health, are significantly associated with frailty and explain substantially more variance than traditional clinical indicators alone.
- Continuous, real-world mobility metrics collected via embedded smartphone sensors provide meaningful insights into functional decline that are not captured by conventional clinic-based frailty assessments.

**What are the implications of the main findings?**

- Smartphone-based monitoring offers a scalable, low-burden approach to community-based frailty assessment and monitoring, with potential to support future longitudinal risk prediction after validation.
- Integrating digital lifelog data into geriatric care pathways can enable proactive intervention, personalized management, and more accurate frailty risk stratification in everyday living environments.

**Abstract:**

Frailty in older adults is a multidimensional syndrome characterized by reduced physiological resilience and heightened vulnerability to adverse outcomes, yet conventional assessments remain largely clinic-based. This study evaluated the feasibility and explanatory utility of smartphone-based digital lifelogs for assessing frailty in community-dwelling older adults. In a prospective observational study, 300 participants (mean age 73.30, SD 5.37 years) from three sites in Seoul, South Korea, used a custom mobile application for two weeks that passively collected sensor-derived gait speed, 30 s sit-to-stand counts, and daily and hourly step counts, alongside self-reported ratings of perceived exertion and subjective health. Frailty Index (FI) scores were computed, and Pearson correlations, hierarchical linear regression, and independent linear regression were applied to examine associations and model explanatory performance. Significant correlations were observed between FI and gait speed, sit-to-stand performance, daily step counts, perceived exertion, and subjective health. Incorporating digital lifelogs significantly improved explained variance in frailty beyond clinical indicators (ΔR^2^ = 0.183), with gait speed and daily step counts emerging as key predictors. A model including only digital lifelogs also significantly associated with frailty (R^2^ = 0.288). These findings suggest that smartphone-based lifelogging offers a feasible, practical, and informative method for two-week monitoring and cross-sectional assessment in community settings.

## 1. Introduction

Frailty is not merely a decline in musculoskeletal function, but a multidimensional syndrome characterized by diminished physiological reserve and impaired resilience to stressors, involving physical, cognitive, psychological, and social vulnerabilities [1]. It often presents as a decline in muscle mass, physical function, cognitive ability, and social engagement, collectively reducing overall function, which significantly impacts quality of life and is closely associated with adverse long-term health outcomes in older adults [2]. Thus, early detection of frailty and identifying high-risk individuals are essential strategies for delaying functional decline and preventing secondary complications [3].

Accurate and sensitive assessment tools are essential for identifying frailty and guiding appropriate interventions. However, most traditional instruments have primarily been designed for hospital-based clinical settings. Among the most widely used tools are the phenotype model of frailty and the cumulative deficit model, which require in-person assessment by trained professionals [4,5,6]. Although these methods offer reliable assessments in controlled environments, implementation in community-dwelling older adult populations remains significantly constrained, especially in areas with limited access to healthcare services [7].

Routine in-person assessments can be challenging for older adults, often resulting in delayed detection of issues and missed opportunities for timely intervention; this highlights the increasing need for simplified and reliable assessment tools that can be used in community settings [8,9]. Tools that allow for self-reporting or remote administration offer greater accessibility, especially in areas with limited healthcare resources [10].

Several frailty assessment tools have been proposed, including the Clinical Frailty Scale (CFS) [9], the Fatigue, Resistance, Ambulation, Illness, and Loss of Weight (FRAIL) scale [11], and the Comprehensive Geriatric Assessment (CGA) [12]. These instruments have demonstrated validity in relation to health outcomes. However, phenotype-based models, such as the Frailty Phenotype (FP), offer quick and easy administration but are primarily effective for frailty classification rather than precise, ongoing monitoring of frailty status. Conversely, cumulative deficit models, such as the Frailty Index (FI) and CGA, while enabling quantitative and detailed frailty monitoring due to their scale data characteristics, are resource-intensive and not practical for community-dwelling settings, particularly for home-based remote monitoring [13,14]. Consequently, a significant gap remains in the routine monitoring of frailty among community-dwelling older adults, indicating a clear need for alternative approaches that balance accuracy, efficiency, and practicality [15,16,17].

Effective management of frailty requires monitoring systems that can continuously and systematically capture daily functional status, fatigue levels, fall risk, and activities of daily living [18,19]. Recent advances in digital health technologies offer promising alternatives for screening and predicting frailty. Various digital tools, including self-administered surveys, wearable sensors, and smartphone-based health applications, provide scalable and user-friendly approaches that facilitate early detection and support self-care practices [19,20,21,22]. Data collected from digital devices is often referred to as digital lifelogs [23,24]. These lifelogs encompass various behavioral and physiological indicators, such as physical activity patterns, sleep cycles, heart rate variability, mobility, and social interactions. Such context-rich data provides valuable insights into an individual’s health status, enabling long-term tracking of frailty-related indicators.

Although several digital frailty assessment tools have been explored [25], research focused on smartphone-based digital lifelogs remains limited, and rigorous validation of these tools has not been widely conducted. Prior studies have utilized smartwatches, wearable accelerometers, fitness trackers, environmental sensors, and smart home Internet of Things (IoT) systems [26,27,28]. For instance, devices such as the ActiGraph GT3X, Fitbit Charge, and Garmin Vivosmart have been used to measure physical activity, sleep, and energy expenditure in older adults, helping to examine associations with frailty status [29,30]. Additionally, smart home sensor networks have been employed to monitor movement, restroom usage frequency, and lighting behavior, which can help identify functional changes relevant to frailty [31].

Smartphones are equipped with various embedded sensors, such as global positioning system (GPS), accelerometers, gyroscopes, ambient light detectors, and, in some models, heart rate monitors [32]. These features enable a practical, non-invasive platform for monitoring frailty without additional hardware, and—given the widespread smartphone adoption among older adults and compatibility with self-report systems and real-time feedback—make smartphone-based monitoring increasingly feasible in daily life [33]. In comparison to wearable devices, using smartphones for data collection offers seamless integration into daily routines and has more significant potential for linking with personalized interventions [34].

Smartphone data is highly relevant to the key components of the frailty phenotype. Measurements obtained from sensors, such as GPS and inertial measurement units (IMUs) [35], can be used to estimate gait speed, sit-to-stand transitions [36,37], physical activity levels [35,36], and sedentary behavior [38,39]. These metrics serve as quantifiable indicators of frailty. However, the use of smartphone-based lifelog data collection in real-world community settings has been limited [40]. Although there is a general agreement on the potential value of digital lifelogs for predicting and monitoring frailty [41], the practical development and validation of such systems have yet to be thoroughly explored.

Hence, the aim of this study was to evaluate the feasibility of using smartphone-based digital lifelogs collected over two weeks to assess frailty status and examine cross-sectional associations with a Frailty Index in community-dwelling older adults.

## 2. Materials and Methods

A prospective observational study was conducted in which digital lifelog data were collected over two weeks; however, frailty was assessed at a single time point, and analyses were conducted as cross-sectional models relating aggregated lifelog features to baseline frailty status.

### 2.1. Recruitment and Sample Size Calculation

Participants were recruited from the department of geriatrics at Asan Medical Center (AMC), the department of endocrinology at Yonsei Severance Hospital (YSH), and the Mokdong Senior Welfare Center (MSWC) in Seoul, South Korea. To be eligible for this study, participants needed to be community-dwelling adults aged 65 years or older. In addition, the following exclusion criteria were applied: (1) current use of systemic corticosteroids during the study period; (2) severe cognitive impairment or diagnosed cognitive deficits that would interfere with participation; (3) ongoing dialysis treatment; (4) presence of ascites due to liver cirrhosis; (5) life expectancy of less than one year as assessed by a physician; and (6) significant difficulty using a smartphone device. The recruitment period ran from 1 September 2023, to 31 December 2023. Each participant who successfully completed this study received a reward of 50,000 won.

Sample size estimation was conducted using G*Power 3.1 software to ensure adequate statistical power for hierarchical linear regression analysis. Assuming a medium effect size (f^2^ = 0.10), an alpha level of 0.05, and a desired power (1−β) of 0.95, with the inclusion of up to 12 predictors (covering demographic, clinical, and digital lifelog variables), the minimum required sample size was calculated to be 184 participants. To accommodate potential dropouts and incomplete data, a final target sample size of 220 participants was established to ensure the robustness and generalizability of the findings.

### 2.2. The Mobile Application

Eligible participants who provided informed consent underwent a thorough CGA that included various components relevant to overall health. Following the assessment, each participant installed a custom-developed mobile application designed to deliver a structured exercise coaching program over two weeks while collecting both self-reported and sensor-derived health data. The application features a ratings of perceived exertion (RPE) scale that allows for assessing exercise intensity using a 15-point system: 1 (normal), 2 (very easy), 3 (unlabeled), 4 (easy), 5 (unlabeled), 6 (somewhat easy), 7 (unlabeled), 8 (slightly hard), 9 (unlabeled), 10 (hard), 11 (unlabeled), 12 (very hard), 13 (unlabeled), 14 (extremely hard), and 15 (maximal exertion). Daily evaluations of subjective health status occurred by responding to the question, “How is health today?” using a 4-point Likert scale: very good, good, not good, or very bad. To assess lower body strength and functional endurance, participants completed the 30 s chair stand test (30 s STS) through the app. Additionally, the application passively collected sensor-based digital lifelogs, which included metrics such as usual gait speed (m/s), 30 s chair stand counts, daily mean step count, and hourly mean step count, through the use of embedded smartphone sensors, including GPS and accelerometers.

### 2.3. Frailty Index

FI was computed using a multidimensional approach that integrates various aspects of an individual’s health status, encompassing medical history, functional abilities, cognitive performance, and physical measurements. This comprehensive assessment aligns with the deficit accumulation model, wherein the presence of multiple health deficits contributes to an increased frailty score.

Medical history was evaluated by tallying the number of specific chronic conditions present in each participant, with a possible range from 0 to 21. Functional status was assessed through the activities of daily living (ADL) and instrumental activities of daily living (IADL) scales. The ADL scale, developed by Katz et al., measures basic self-care tasks such as bathing, dressing, and feeding [42]. The IADL scale, introduced by Lawton and Brody, evaluates more complex activities necessary for independent living, including managing finances, medication, and transportation [43].

Physical functioning was further examined using Nagi’s model of disablement, which conceptualizes the progression from pathology to disability through stages of impairment and functional limitation [44]. Additionally, the Rosow-Breslau Scale was employed to assess mobility-related tasks, such as walking up stairs and performing heavy housework, providing insight into the participants’ physical capabilities [45].

Weight-related factors were incorporated by assigning points for significant weight loss (over 4.5 kg within the past year), skeletal muscle mass (SMM), and a body mass index (BMI) of ≤18.5 kg/m^2^. The aforementioned variables were measured using the InBody 770 and S10 (InBody Co., Ltd., Seoul, Republic of Korea). Cognitive function was evaluated using the Mini-Cog test, with scores converted to a frailty scale: a score of 5 equated to 0 points, 4 to 0.3 points, scores between 1 and 3 to 0.7 points, and a score of 0 to 1 point [46].

Physical performance was measured using components of the short physical performance battery (SPPB). To measure the SPPB test results, a toolkit consisting of sensors and software (Dyphi, Daejeon, Republic of Korea) was used, as validated and reported in previous studies [47,48]. The sit-to-stand test was scored based on completion time, with longer durations indicating higher frailty. Gait speed was similarly categorized, with slower speeds corresponding to increased frailty points [49]. Handgrip strength was assessed using gender-specific thresholds, recognizing its correlation with overall muscle strength and functional status [50]. The Handgrip strength was assessed twice using the Jamar Plus+ Digital Dynamometer, and the maximum value of the two trials was recorded.

The cumulative deficit score was divided by the total number of possible deficits (maximum = 50) to derive a normalized FI ranging from 0 to 1, consistent with the standard deficit accumulation approach [5]. In instances of missing deficit items, the denominator was adjusted accordingly, such that the FI represented the proportion of deficits present among all non-missing items, in accordance with established CGA-FI methodology. This methodology is consistent with the comprehensive geriatric assessment–frailty index (CGA-FI), which emphasizes a holistic evaluation of older adults’ health [51].

### 2.4. Statistical Analysis

Descriptive statistics were reported as means and standard deviations (SD) for all continuous variables. Pearson correlation coefficients were calculated to investigate the relationships between each digital lifelog variable and the Frailty Index. Hierarchical linear regression analysis was conducted to determine whether digital lifelog data significantly enhanced explanatory performance beyond conventional clinical indicators. Initially, a baseline regression model was constructed, incorporating demographic and anthropometric variables such as age, sex, height, weight, SMM, body fat percentage, and SPPB scores. In the subsequent model, digital lifelog variables showing significant correlations—excluding hourly mean steps, which exhibited no significant association—were introduced to assess their incremental predictive contribution. Additionally, a separate linear regression analysis was conducted using only digital lifelog variables to evaluate their independent predictive capacity regarding the FI.

Clusters underwent additional filtering to eliminate instantaneous speeds below 0.3 m/s or above 2.0 m/s. Clusters representing movements lasting fewer than 10 min or with radii less than 100 m were excluded. The remaining clusters were manually verified to remove trajectories located in unsuitable environments such as bodies of water, highways, indoor spaces, or areas prone to GPS signal distortion, including mountainous or densely vegetated park regions. Instantaneous speeds between GPS points within validated clusters were computed and smoothed using a 50-point moving average, and the mean of these smoothed speeds constituted the participants’ usual gait speed.

Step counts were collected via the smartphone’s internal pedometer sensor, from which daily and hourly mean step counts were computed based on timestamps. Performance on the 30 s sit-to-stand (30 s STS) test was assessed using accelerometer data actively gathered during an in-app instructed chair-stand task. Further digital lifelog variables included self-reported RPE and daily subjective health assessments recorded through the application’s interface. All statistical analyses and preprocessing were conducted using Python 3.11.

### 2.5. Measurements

Digital lifelog data were systematically collected over two weeks using a pilot mobile application, capturing GPS data, pedometer-based step counts, and accelerometer data from the smartphone’s inertial measurement unit (IMU). GPS-based activity data were obtained via Google’s Detected Activity API, categorizing participant activities such as IN_VEHICLE, ON_FOOT, and STILL. Usual gait speed was calculated through a rigorous multi-step refinement process. Initially, raw GPS data, including latitude, longitude, time stamps, and activity states, were documented. Only data points specifically labeled as “Walking” were retained for further analysis. Consecutive GPS points separated by intervals no greater than five minutes were grouped into clusters, excluding those containing fewer than 100 data points.

For transparency and reproducibility, detailed definitions, ranges, units, and coding schemes for all demographic, clinical, physical performance, and digital lifelog variables used in the analyses are provided in Table S1.

## 3. Results

**Figure 1:**
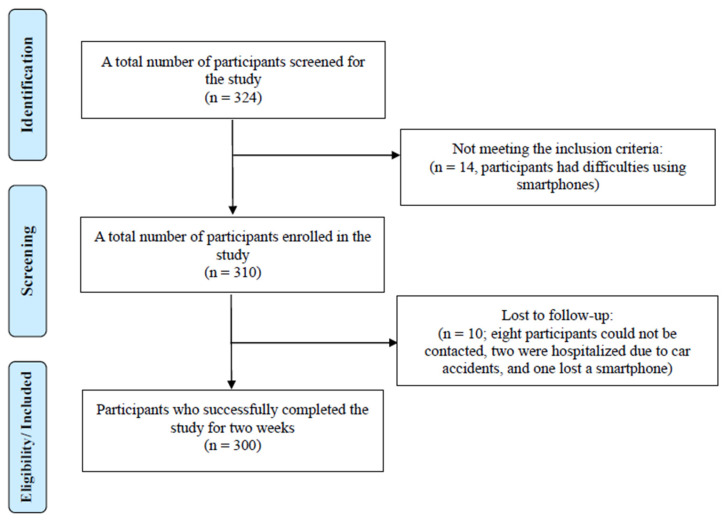
Flow chart of the inclusion and exclusion process.

A total of 324 older adults were recruited and agreed to participate in this study. However, 14 individuals were unable to take part due to difficulties using smartphones, resulting in 310 participants being enrolled. Before this study concluded, 10 participants were lost to follow-up: eight could not be reached three days after this study started, two were hospitalized due to car accidents, and one lost a smartphone. Ultimately, 300 older adults were included in this study. The details of the inclusion and exclusion process are provided in Figure 1.

### 3.1. Participant Characteristics

The demographic characteristics of participants from three different recruitment sites are summarized in Table 1. Overall, the mean age of participants was 73.30 (SD 5.37) years. Those from the MSWC had a slightly higher mean age of 75.41 (SD 5.21) years compared to participants from the YSH, who had a mean age of 71.78 (SD 5.24) years, and AMC, with a mean age of 74.11 (SD 4.18) years. Furthermore, the proportion of female participants was highest at YSH, which had 90.68% female participants, followed by MSWC at 67.96% and AMC at 61.11%.

Participants from AMC had a significantly higher average weight of 60.50 (SD 13.92) kg compared to those from the YSH, whose average weight was 55.38 (SD 7.29) kg. The mean BMI values were highest among participants from the MSWC, averaging 25.05 (SD 3.33) kg/m^2^, followed by the YSH at 22.95 (SD 2.73) kg/m^2^ and AMC at 23.39 (SD 3.21) kg/m^2^. Furthermore, the prevalence of participants who experienced significant weight loss (more than 4.5 kg within a year) was greater in AMC, with 16.67%, compared to the YSH at 6.83% and the MSWC at 9.71%. When assessing cognitive function using the Mini-Cog test, the AMC had the highest average score (4.58 ± 0.60), followed by the MSWC (4.25 ± 0.89), and then YSH (3.78 ± 1.13).

In terms of physical composition, SMM was highest at AMC, with an average of 24.17 (SD 7.41) kg, followed by the MSWC at 22.39 (SD 4.51) kg, and then YSH at 20.87 (SD 13.57) kg. Moreover, handgrip strength was significantly stronger among participants from AMC, averaging 24.51 (SD 10.25) kg, and those from the MSWC, averaging 24.91 (8.57) kg, compared to participants at YSH, with an average of 18.56 (4.68) kg.

Physical performance was assessed using the SPPB, yielding a mean total score of 11.17 (SD 1.52) across all participants, with minor variations observed between locations. The average gait speed was highest at AMC, measuring 1.16 (SD 0.62) m/s, compared to the MSWC at 1.09 (SD 0.23) m/s and YSH at 0.92 (SD 0.26) m/s. In the 5-times sit-to-stand test, participants from YSH recorded the fastest completion time at 3.58 (SD 0.67) seconds, followed by AMC at 3.72 (SD 0.57) seconds and the MSWC at 3.87 (SD 0.44) seconds.

The normalized FI indicated a relatively low to mild level of frailty in the study population, with a mean FI of 0.091 (SD 0.069). Among sites, the highest mean FI was observed in YSH at 0.109 (0.072), followed by AMC at 0.085 (0.060), while MSWC exhibited the lowest frailty levels at 0.065 (0.057).

### 3.2. Characteristics of the Collected Digital Lifelogs

A total of six digital lifelog variables were collected and analyzed, which included both sensor-based and self-report measures. Among the sensor-derived metrics, the usual gait speed was calculated for 94 participants, yielding a mean value of 1.12 (SD 0.13) m/s. The 30 s sit-to-stand test, completed by 253 participants, resulted in an average of 17.36 (SD 5.12) repetitions. Data on daily mean step counts, collected from pedometer readings, was available for 290 participants and showed considerable variability, with a mean of 3343.93 (SD 3049.45) steps. Additionally, hourly mean step counts derived from timestamped pedometer data were also available for 290 participants, averaging 759.79 (SD 590.18) steps.

Self-reported data collected through the mobile application included RPE and subjective health status. The RPE, reported by 248 participants, had a mean score of 5.88 (SD 2.17) on a 15-point scale, indicating a moderate perceived intensity during the exercise program. Subjective health status, rated by 255 participants on a 4-point Likert scale, had a mean score of 2.05 (SD 0.42), suggesting a generally positive self-perception of daily health. These findings demonstrate the feasibility and variability of digital lifelog collection within a community-based population of older adults.

### 3.3. Factors Correlated Between FI and Digital Lifelogs

Pearson’s correlation analyses were performed to investigate the relationships between the normalized FI (0–1 scale) and various digital lifelog variables (Table 2). Usual gait speed exhibited the strongest negative correlation with the FI (r = −0.370, *p* < 0.001), indicating that slower walking speed is significantly associated with a higher level of frailty. Similarly, the number of repetitions in the 30 s sit-to-stand test showed a significant inverse relationship with the FI (r = −0.224, *p* < 0.001), suggesting that reduced lower-limb strength is related to increased frailty. The daily mean step count also presented a weak but statistically significant negative correlation with the FI (r = −0.119, *p* = 0.042). In contrast, the hourly mean step count approached statistical significance (r = −0.113, *p* = 0.055) but did not reach the conventional threshold for significance.

The results showed a positive correlation between the RPE and the FI (r = 0.135, *p* = 0.034); this suggests that individuals with higher levels of frailty reported experiencing greater exertion during physical activities. Furthermore, a significant positive correlation was found with subjective health status (r = 0.232, *p* < 0.001), indicating that participants with higher frailty scores tended to rate their health more negatively. Overall, these findings highlight the importance of both sensor-based and self-reported digital lifelog indicators in reflecting the physiological and perceptual characteristics associated with frailty in community-dwelling older adults.

### 3.4. Frailty Modeling Using Digital Lifelogs

A hierarchical linear regression analysis was conducted to determine whether digital lifelog variables significantly enhanced the explanatory modeling of frailty, as measured by the FI (Table 3). In Step 1, a model was evaluated that included traditional indicators of frailty phenotype, such as age, sex, height, weight, SMM, body fat percentage, and the SPPB score. This initial model did not show statistical significance, F(7, 64) = 1.53, *p* > 0.05, and accounted for 14.5% of the variance in the FI (R^2^ = 0.145).

In Step 2, digital lifelog variables were incorporated into the model, including usual gait speed, the number of 30 s sit-to-stand counts, daily mean step count, RPE, and subjective health status; this inclusion significantly improved the overall model fit, F(12, 59) = 2.36, *p* < 0.05, and resulted in a notable change in R^2^ (ΔR^2^ = 0.183), increasing the explained variance to 32.8%. Among the digital lifelog predictors, usual gait speed (β = −0.242, *p* = 0.047) and daily mean step count (β = 0.272, *p* = 0.025) significantly contributed to the prediction of frailty. Subjective health status approached significance (β = 0.254, *p* = 0.068), while the other variables, including 30 s STS counts and RPE, did not reach statistical significance (Table 4).

To evaluate the independent explanatory value of the digital lifelog variables, a separate linear regression model was conducted using only these parameters (Table 5). The model was statistically significant, with F(5, 66) = 5.35, *p* < 0.001, and an R^2^ of 0.288; this indicates that the digital lifelog data accounted for 28.8% of the variance in the FI. The variance inflation factors (VIFs) for each digital lifelog within the models ranged from 1 to 2, indicating a low likelihood of problematic multicollinearity. Within this model, the following variables emerged as significant predictors: usual gait speed (β = −0.300, *p* = 0.007), daily mean steps (β = 0.288, *p* = 0.012), and subjective health status (β = 0.317, *p* = 0.007). Conversely, the 30 s sit-to-stand counts (*p* = 0.168) and the RPE (*p* = 0.345) did not demonstrate significant associations with the FI.

To further examine the robustness of these findings, a series of sensitivity analyses were conducted. First, we re-estimated the complete hierarchical model after removing daily mean steps (Table S2). This model remained statistically significant (R^2^ = 0.267, F = 1.95, *p* < 0.05), and usual gait speed continued to show a significant negative association with the FI (β = −0.263, *p* = 0.038), whereas 30 s STS counts, RPE, and subjective health status remained non-significant.

Next, we fitted a complementary model excluding usual gait speed while retaining daily mean steps and the other digital lifelog variables (Table S3). This model was also statistically significant (R^2^ = 0.281, F = 2.09, *p* < 0.05). In this specification, daily mean steps remained a significant positive predictor of FI (β = 0.290, *p* = 0.019), and subjective health status emerged as a significant positive correlate (β = 0.286, *p* = 0.045), whereas 30 s STS counts and RPE again did not reach statistical significance.

To assess the independent effects of each digital lifelog indicator, additional models were estimated in which each digital variable was entered one at a time, along with the covariates (Table S4). When usual gait speed was added individually, the model explained 22.3% of the variance in FI (R^2^ = 0.223, *p* < 0.05), and gait speed remained a significant negative predictor (β = −0.298, *p* = 0.015). Models including only daily mean steps (R^2^ = 0.208, model *p* ≈ 0.06; β = 0.256, *p* = 0.031) and only subjective health status (R^2^ = 0.197, model *p* ≈ 0.08; β = 0.246, *p* = 0.049) also showed significant or borderline-significant associations with FI, whereas models including only 30 s STS counts or only RPE were not significant.

Finally, a robust regression using Huber’s T estimator was performed to evaluate the influence of potential outliers (Table S5). The robust model demonstrated a comparable level of explained variance (R^2^ = 0.320; χ^2^ = 26.67, *p* < 0.05), and daily mean steps remained a significant positive predictor of FI (*p* = 0.023). Usual gait speed showed a similar negative trend as in the OLS models, although it did not reach conventional significance (*p* = 0.072), and the remaining predictors were non-significant. Overall, the robust regression results support the general pattern observed in the standard linear models, suggesting that a small number of influential observations does not drive the main findings.

## 4. Discussion

This study demonstrated that smartphone-based digital lifelogs are a feasible and valuable method for assessing frailty in older adults living in the community. Over the course of two weeks, participants used a custom mobile application that passively recorded sensor metrics and actively collected self-reports. Hierarchical regression analyses revealed that incorporating these digital lifelog measures significantly improved the cross-sectional model’s explanatory performance beyond traditional clinical predictors alone, highlighting unique insights offered by continuous, ambient data collection.

Importantly, older participants successfully engaged with the smartphone application over a two-week period, demonstrating the practicality of continuous digital data collection for this demographic. Participants generally showed good adherence to carrying the device and completing self-reports, indicating that even seniors with limited technology experience can effectively use health apps with adequate support. This finding aligns with a recent feasibility study conducted in Japan, where senior citizens used smartphones for lifelogging without additional stress, even as first-time users [52]. Furthermore, smartphone ownership and digital literacy among older adults are steadily increasing, making these mobile health approaches more realistic on a larger scale [53].

Notably, usual gait speed and daily step count emerged as strong independent indicators of frailty status [54,55]. While clinical indices, such as the SPPB, measure gait speed under artificial, instructed conditions, digital lifelog methods utilizing GPS-based ambient monitoring potentially capture a more authentic representation of typical gait speed. This ambient measurement approach provides unique value by assessing real-world mobility patterns beyond the controlled clinic environment. Additionally, self-rate health, captured through the app’s daily surveys, made a significant contribution to the model, highlighting the importance of subjective health perception in frailty assessment. Overall, these findings highlight how everyday mobility patterns and self-assessed well-being are often overlooked in brief clinical encounters, offering critical insights into an individual’s risk of frailty [56,57].

Moreover, digital lifelog indicators may help identify individuals who are pre-frail but have not yet reached clinical frailty thresholds and are, therefore, at risk [58,59]. These indicators effectively capture subtle changes in gait variability, mobility consistency, and self-reported wellness over time [60]. The incorporation of digital lifelog variables significantly improved explanatory models for frailty, increasing explained variance from 14.5% with clinical indicators alone to 32.8%, underscoring their substantial added value. Additionally, specific digital lifelog parameters, such as gait speed (β = −0.300, *p* = 0.007), daily step count (β = 0.288, *p* = 0.012), and subjective health perception (β = 0.317, *p* = 0.007), significantly contributed individually to explaining frailty levels, reinforcing their unique predictive power. These findings align with recent studies highlighting wearable-derived gait metrics as effective predictors of frailty and health risks among older populations. Osuka et al. reported significant associations between wearable-derived gait variability, maximum gait speed, and reduced daily step count with frailty in a large cohort of older adults [61]. Similarly, Chan et al. demonstrated that digital gait biomarkers from wrist sensor data effectively predict future injurious falls, further supporting the broader value of real-world mobility tracking [62]. Hence, continuous, unobtrusive mobility monitoring can detect subtle functional declines and enhance frailty status characterization beyond episodic clinical assessments.

Interestingly, although initial associations existed for RPE and sit-to-stand counts with frailty, these measures did not maintain significance in multivariate analyses, potentially reflecting that daily mobility and subjective wellness are more representative of regular functioning than episodic performance assessments [54,63]. Furthermore, the limited variability or sensitivity of standard physical performance tests, such as the SPPB, within community-dwelling populations may also explain the lack of significance in predictive models.

The current study further aligns with and extends previous evidence emphasizing the importance of real-world gait and activity monitoring. Smartphone-derived gait parameters have effectively differentiated between frail and non-frail older adults, capturing functional aspects that are not easily assessed through traditional clinical tests or questionnaires [64]. Recent research has demonstrated a significant enhancement in frailty prediction by combining wearable-measured walking speed and daily activity metrics with clinical data, achieving an AUC of 0.93 compared to clinical measures alone. Digital lifelog data similarly highlighted the critical role of real-life walking speed and daily activity levels in identifying frailty [65]. Continuous tracking of such gait-related indicators through smartphones provides a sensitive means of detecting early functional decline. Moreover, combining objective sensor data with subjective health ratings captures the multidimensional interplay of physical and psychosocial factors affecting frailty more effectively [66,67].

Considering the limited time older adults spend in clinical settings compared to their overall daily lives, digital lifelogging represents a practical method for characterizing and monitoring quantitative frailty status within community environments. Preliminary analyses suggest that digital lifelog data alone may closely track clinical assessment indicators such as the CGA-FI, highlighting its potential utility independent of clinical measures. Previous studies support the use of mobile devices as non-invasive, cost-effective, and user-friendly tools for remote frailty monitoring [64]. Collecting lifelog data in community settings may enable earlier detection of subtle declines preceding clinical frailty, opening new possibilities in preventive geriatrics. Real-time frailty monitoring could facilitate early interventions, personalized care planning, and automated caregiver or clinician alerts [68]. Hence, these findings underscore both the unique predictive value and practical feasibility of smartphone-based digital lifelogs in frailty assessment among community-dwelling older adults.

This study has several strengths, including its multi-center design and rigorous validation against comprehensive geriatric assessments. It also benefits from real-world data collection across diverse urban environments. By using embedded smartphone sensors instead of specialized wearables, the methodology improves scalability and minimizes participant burden—key advantages for community-based implementation. The two-week monitoring period provided sufficient data granularity to detect subtle functional changes, thereby addressing the limitations of single-time point assessments that are common in traditional frailty research.

Several limitations of this study should be acknowledged. First, the generalizability of findings is limited to community-dwelling older adults who own and can effectively use smartphones. While all participants demonstrated a basic level of digital literacy, this may not accurately represent the broader older adult population, particularly those who are more disadvantaged, reside in institutions or lack confidence in using technology. Recent survey data indicate that although smartphone ownership is widespread among older adults, only a minority feel comfortable using mobile applications, highlighting a significant gap in digital literacy [69]. Additionally, this study was conducted within a specific geographic and cultural context, which may restrict the applicability of results to other regions or populations, especially among very old adults, those with advanced frailty, or individuals without access to modern smartphones. In addition, although the sensitivity analyses suggested that the pattern of regression coefficients was broadly maintained across alternative model specifications and robust estimation, the effective sample size for the multivariable models was relatively small and the predictive performance deteriorated under 5-fold cross-validation. These findings indicate that the predictive value of the digital lifelog variables should be regarded as preliminary and warrants replication and external validation in larger and more diverse samples.

Second, the reliability of the collected lifelog data depended on consistent user engagement and proper device usage. Instances in which participants forgot to carry smartphones or failed to interact with the app as instructed could have led to missing or inaccurate data, potentially weakening the observed associations. Technical factors, such as phone placement and calibration, may have also introduced measurement errors despite efforts to address these issues through data processing and validation. Furthermore, the two-week monitoring period provides only a limited snapshot of participant behaviors and may not fully capture the dynamic, long-term nature of frailty. Longer-term studies are needed to determine whether extended monitoring can improve predictive accuracy and better track changes in frailty status over time.

## 5. Conclusions

This study demonstrated that smartphone-based digital lifelogs collected over two weeks are viable and informative for the cross-sectional evaluation and modeling of frailty status in community-dwelling older adults. Given the modest explained variance and cross-sectional design, these findings should be interpreted as exploratory and hypothesis-generating, demonstrating feasibility and associative utility rather than definitive predictive accuracy. Digital lifelog features may represent antecedents of frailty, consequences of frailty, or parallel manifestations of shared underlying processes. The research indicated that key metrics derived from sensors, such as usual gait speed and daily average steps, in conjunction with individuals’ self-reported health status, are strong correlations of the FI, underscoring the utility of mobile technology in this context. Importantly, the integration of these digital lifelogs significantly improved predictive accuracy compared to traditional clinical indicators, addressing a critical gap in current frailty screening methods. These findings highlighted the transformative potential of digital health in gerontology, paving the way for the development of accessible, scalable, and non-invasive tools that facilitate early intervention and personalized health management, ultimately enhancing the quality of life and delaying functional decline in aging populations. Future research should validate these digital markers and employ longitudinal designs to assess effectiveness in predicting future frailty trajectories and clinical outcomes.

## Figures and Tables

**Table 1:** General characteristics of the study participants.

Category	YSH (*n* = 161)	AMC (*n* = 36)	MSWC (*n* = 103)	Total (*n* = 300)
Age (year)	71.78 ± 5.24	74.11 ± 4.18	75.41 ± 5.21	73.30 ± 5.37
Female (%)	90.68% (146)	61.11% (22)	67.96% (70)	79.33% (238)
Height (cm)	155.37 ± 6.09	160.63 ± 9.57	157.79 ± 8.08	156.83 ± 7.49
Weight (kg)	55.38 ± 7.29	60.50 ± 13.92	62.41 ± 9.80	58.40 ± 9.73
Medical history (*n*)	3.36 ± 2.39	2.56 ± 1.81	1.75 ± 1.51	2.71 ± 2.19
ADL	0.15 ± 0.45	0.00 ± 0.00	0.08 ± 0.27	0.11 ± 0.37
IADL	0.05 ± 0.37	0.08 ± 0.50	0.10 ± 0.38	0.07 ± 0.39
Nagi	0.39 ± 78	0.25 ± 0.65	0.39 ± 0.69	0.37 ± 0.74
Rosow	0.16 ± 0.43	0.17 ± 0.51	0.12 ± 0.40	0.15 ± 0.43
Weight loss over 4.5 kg within the year (%)	6.83%	16.67%	9.71%	9.00%
BMI ≤ 18.5 kg/m^2^ (%)	2.48%	11.11%	1.96%	3.33%
Mini-Cog	3.78 ± 1.13	4.58 ± 0.60	4.25 ± 0.89	4.04 ± 1.04
BMI (kg/m^2^)	22.95 ± 2.73	23.39 ± 3.21	25.05 ± 3.33	23.72 ± 3.15
SMM (kg)	20.87 ± 13.57	24.17 ± 7.41	22.39 ± 4.51	21.79 ± 10.64
Fat (%)	32.19 ± 6.74	25.04 ± 6.95	32.59 ± 7.42	31.46 ± 7.38
Right arm muscle (kg)	1.80 ± 0.35	2.33 ± 0.78	2.13 ± 0.55	1.98 ± 0.53
Left arm muscle (kg)	1.80 ± 0.35	2.32 ± 0.75	2.11 ± 0.55	1.97 ± 0.52
Right leg muscle (kg)	5.51 ± 0.93	6.69 ± 2.11	6.22 ± 1.47	5.90 ± 1.38
Left leg muscle(kg)	5.50 ± 0.90	6.66 ± 2.04	6.19 ± 1.45	5.87 ± 1.35
Handgrip strength (kg)	18.56 ± 4.68	24.51 ± 10.25	24.91 ± 8.57	21.45 ± 7.67
SPPB	11.09 ± 1.52	10.44 ± 1.80	11.54 ± 1.28	11.17 ± 1.52
Side-by-side (s)	10.00 ± 0.00	10.00 ± 0.00	10.00 ± 0.00	10.00 ± 0.00
Semi-tandem (s)	9.63 ± 1.75	9.13 ± 2.37	9.85 ± 1.13	9.65 ± 1.67
Tandem (s)	9.00 ± 2.70	7.61 ± 3.64	9.01 ± 2.70	8.85 ± 2.84
6 m gait speed (m/s)	0.92 ± 0.26	1.16 ± 0.62	1.09 ± 0.23	1.01 ± 0.33
5× sit-to-stand (s)	3.58 ± 0.67	3.72 ± 0.57	3.87 ± 0.44	3.70 ± 0.60
Frailty Index (normalized, 0–1)	0.109 ± 0.072	0.085 ± 0.060	0.065 ± 0.057	0.091 ± 0.069

**Table 2:** Characteristics of digital lifelogs.

Digital Lifelogs	*n*	Mean (SD)
Sensor-based Digital Lifelog		
Usual gait speed (m/s)	94	1.12 (0.13)
30 s STS counts	253	17.36 (5.12)
Daily mean steps	290	3343.93 (3049.45)
Hourly mean steps	290	759.79 (590.18)
Self-report-based Digital Lifelogs		
RPE	248	5.88 (2.17)
Subjective health status	255	2.05 (0.42)

**Table 3:** Pearson’s correlations between FI and digital lifelogs.

Set	*n*	Pearson’s r	*p*-Value	95% CI
Frailty Index—Usual gait speed	94	−0.370 ***	<0.001	[−0.533, −0.181]
Frailty Index—30 s STS counts	253	−0.224 ***	<0.001	[−0.338, −0.104]
Frailty Index—Daily mean steps	290	−0.119 *	0.042	[−0.231, −0.004]
Frailty Index—Hourly mean steps	290	−0.113	0.055	[−0.225, 0.002]
Frailty Index—RPE	248	0.135 *	0.034	[0.011, 0.255]
Frailty Index—Subjective health status	255	0.232 ***	<0.001	[0.112, 0.345]

* p < 0.05, *** *p* < 0.001.

**Table 4:** Results of hierarchical linear regression with normalized FI as the dependent variable.

Predictors	Step 1 (Frailty Phenotypes)					Step 2 (Add Digital Lifelogs)				
	B	SE	β	*p*	VIF	B	SE	β	*p*	VIF
Age (year)	−0.001	0.002	−0.040	0.763	1.082	0.0001	0.002	0.009	0.945	1.481
Sex (1 = female, 0 = male)	0.008	0.033	0.055	0.812	2.384	0.007	0.034	0.050	0.834	4.894
Height (cm)	−0.002	0.002	−0.267	0.315	3.436	−0.001	0.002	−0.172	0.489	5.297
Weight (kg)	0.002	0.002	0.289	0.357	3.316	0.001	0.002	0.112	0.727	8.780
SMM (kg)	0.0001	0.003	0.008	0.974	1.163	0.001	0.003	0.09	0.704	4.768
Fat (%)	0.001	0.002	0.159	0.521	2.711	0.001	0.002	0.119	0.614	4.742
SPPB	−0.006	0.007	−0.109	0.401	1.042	−0.001	0.007	−0.026	0.844	1.447
Usual gait speed (m/s)	–	–	–	–		−0.116	0.057	−0.242	0.047 *	1.234
30 s STS counts	–	–	–	–		−0.001	0.001	−0.088	0.472	1.277
Daily mean steps	–	–	–	–		0.000	0.000	0.272	0.025 *	1.197
RPE	–	–	–	–		−0.001	0.004	−0.026	0.859	1.807
Subjective health status	–	–	–	–		0.036	0.019	0.254	0.068	1.612
R^2^	0.145					0.328				
F for Model	1.53					2.36 *				
F for change in R^2^	-					3.16 *				

* p < 0.05. ΔR^2^ = 0.183; F-change (5, 59) = 3.16, *p* < 0.05; Overall Step 2 model: F(12,59) = 2.36, *p* < 0.05.

**Table 5:** Regression coefficients of digital lifelogs associated with normalized FI.

Predictors	B_Normalized	SE_Normalized	β	T	*p*-Value	95% CI	VIF
Usual gait speed	−0.144	0.051	−0.3	−2.81	0.007 **	[−12.30, −2.10]	1.050
30 s STS counts	−0.002	0.001	−0.179	−1.395	0.168	[−0.20, 0.05]	1.009
Daily mean steps	0.000	0.000	0.288	2.596	0.012 *	[0.00005, 0.00055]	1.143
RPE	−0.003	0.003	−0.105	−0.952	0.345	[−0.50, 0.15]	1.203
Subjective health status	0.045	0.016	0.317	2.76	0.007 **	[0.60, 3.85]	1.200

* p < 0.05, ** *p* < 0.01. R^2^ = 0.288; F(5, 66) = 5.35, *p* < 0.001. K-fold Cross-validation (K = 5) R^2^ = −0.0751 (SD = 0.234).

## Data Availability

The data presented in this study are available from the corresponding author upon reasonable request. Due to confidentiality agreements, supporting data can be provided only to bona fide researchers and will require a non-disclosure agreement.

## Supplementary Materials

The following supporting information can be downloaded at: https://www.mdpi.com/article/10.3390/s26010215/s1, Table S1. Definitions, coding, and measurement properties of study variables. Table S2. Regression results for model excluding daily mean steps. Table S3. Regression results for model excluding usual gait speed. Table S4. Regression results for model each digital lifelogs individually. Table S5. Robust regression (Huber’s T) results.

## Abbreviations

The following abbreviations are used in this manuscript:

ADL	Activities of Daily Living
AWGS	Asian Working Group for Sarcopenia
BMI	Body Mass Index
CGA	Comprehensive Geriatric Assessment
CGA-FI	Comprehensive Geriatric Assessment–Frailty Index
CFS	Clinical Frailty Scale
FI	Frailty Index
FP	Frailty Phenotype
FRAIL	Fatigue, Resistance, Ambulation, Illness, and Loss of Weight
GPS	Global Positioning System
IADL	Instrumental Activities of Daily Living
IMU	Inertial Measurement Unit
IoT	Internet of Things
RPE	Ratings of Perceived Exertion
SD	Standard Deviation
SE	Standard Error
SMM	Skeletal Muscle Mass
SPPB	Short Physical Performance Battery
30 s STS	30-second Sit-to-Stand Test
