# Supplementary material for: Assessment of Frailty in Community-Dwelling Older Adults Using Smartphone-Based Digital Lifelogging: A Multi-Center, Prospective Observational Study
Source: Sensors (Basel). 2025 Dec 29;26(1):215. doi: 10.3390/s26010215 (PMC12788275; doi:10.3390/s26010215)
Supplement: Supplementary file 1 [file sensors-26-00215-s001.zip › sensors-4032561-supplementary.pdf]

Table S1. Definitions, coding, and measurement properties of study variables.

Variable	Definition	Range	Units
id	Participant identifier	n/a	count
org	Recruiting institution	n/a	n/a
sex	Sex (female or male)	n/a (Sex was coded as a dummy variable (female = 1, male = 0) in the regression models.)	n/a
age	Age (years)	over 65	n/a
height	Height (cm)	over 0	cm
weight	Weight (kg)	over 0	kg
medical_history	Number of conditions present among: Angina; Anxiety disorder; Arthritis; Asthma; Atrial fibrillation/flutter; Cancer within 5 years; Chronic kidney disease (eGFR < 60); COPD; Coronary artery disease; Degenerative spine disease; Dementia; Depression; Diabetes; Fall within the past year; Heart failure; Hypertension; Myocardial infarction; Peripheral vascular disease; Sensory impairment; Stroke/TIA; and use of 5 or more prescription drugs.	0–21	count
adl_total	Number of basic ADL items (dressing, washing face, bathing, eating, transferring, toileting, continence) for which the participant cannot perform independently and requires assistance.	0–7	count
iadl_total	Number of IADL items (using a smartphone to make calls/send texts, using public transportation, shopping, preparing meals, doing housework, managing medications, managing finances) for which the participant cannot perform independently and requires assistance.	0–7	count
nagi_total	Number of Nagi disability items (bending or kneeling/squatting, reaching above shoulder level, pushing or pulling heavy objects, lifting and carrying an object of about 4.5 kg, writing or handling small objects) that the participant is unable to perform.	0–5	count
rosow_total	Number of Rosow–Breslau items (cleaning windows/walls/floors, climbing up and down two flights of stairs, walking 800 m) for which the participant requires assistance.	0–3	count
nutritional_status_1	Weight loss ≥ 4.5 kg during the past year (yes/no).	0–1	count
nutritional_status_2	BMI < 18.5 kg/m^2 (yes/no).	0–1	count
nutritional_status_3	Serum albumin < 3.5 g/L on blood test (yes/no).	0–1	count
minicog	Total score on the Mini-Cog test.	0–5	score
grip	Maximum value from two trials of dominant-hand grip strength.	over 0	kg
sppb_gait_speed	5 m gait speed measured in the SPPB test.	over 0	meter/sec
sppb_gait_speed_score	SPPB scoring value corresponding to the 5 m gait speed.	0–4	score
sppb_balance_side-by-side	Holding time in the side-by-side stance.	0–10	sec
sppb_balance_semi-tandem	Holding time in the semi-tandem stance.	0–10	sec

sppb_balance_tandem	Holding time in the tandem stance.	0–10	sec
sppb_balance_score	SPPB static balance score.	0–4	score
sppb_5×_chairstand	Time required to complete five chair stands in the SPPB test.	0–60	sec
sppb_5×_chairstand_score	Score assigned based on the time required to complete five chair stands.	0–4	score
sppb_total_score	Total SPPB score (sum of sppb_gait_speed_score, sppb_balance_score, and sppb_5×_chairstand_score).	0–12	score
bmi	Body mass index.	over 0	kg/m^2
upper_rt	Right upper-limb muscle mass.	over 0	kg
upper_lt	Left upper-limb muscle mass.	over 0	kg
lower_rt	Right lower-limb muscle mass.	over 0	kg
lower_lt	Left lower-limb muscle mass.	over 0	kg
muscle	Total skeletal muscle mass.	over 0	kg
fat	Body fat percentage.	0–100	%
wbc	White blood cell count.	over 0	10^3/μL
hemoglobin	Hemoglobin level.	over 0	g/dL
glucose	Blood glucose level.	over 0	mg/dL
albumin	Serum albumin level.	over 0	g/dL
creatine	Serum creatinine level.	over 0	mg/dL
sodium	Serum sodium level.	over 0	mmol/L
crp	C-reactive protein (CRP) level.	over 0	mg/dL
frailty_index	Frailty Index (cumulative deficit index, 0–1).	0–1	index
adherence_rate	Adherence rate to app use.	0–1	percentage
30 sts	Number of sit-to-stand repetitions completed in 30 s, recorded in the app.	n/a	count
step_daily	Mean daily step count.	over 0	steps
step_hourly	Mean hourly step count.	over 0	steps
gait_cadence	Mean number of steps per minute (gait cadence).	over 0	steps
gait_speed	Usual gait speed in daily life estimated from GPS data.	over 0	meter/s
rpe_mean	Mean rating of perceived exertion (RPE) recorded in the app.	1–15	score
subjective_health	Self-rated health score recorded in the app. 1 = Very good, 2 = Good, 3 = little bad, 4 = extremely bad	1–4	score

Table S2. Regression results for model excluding daily mean steps.

**Predictors**	**B**	**SE**	**β**	***p*-Value**	**VIF**
Age (year)	−0.0002	0.1351	−0.0196	0.8854	1.47
Sex (1 = female, 0 = male)	0.0123	0.2462	0.0855	0.7296	4.87
Height (cm)	−0.0016	0.2563	−0.2011	0.4357	5.28
Weight (kg)	0.0014	0.328	0.2000	0.5444	8.65
SMM (kg)	0.0008	0.2432	0.0638	0.7939	4.76
Fat (%)	0.0009	0.2426	0.0993	0.6839	4.74
SPPB	−0.0024	0.1339	−0.0425	0.752	1.44
Usual gait speed (m/s)	−0.1262	0.1235	−0.2629	0.0375 *	1.23
30 s STS counts	−0.001	0.126	−0.0913	0.4716	1.28
RPE	0.0022	0.1427	0.0763	0.5946	1.64
Subjective health status	0.0236	0.1359	0.1664	0.2258	1.49
R^2	0.267				
F for Model	1.95 *				

* $p < 0.05$.

Table S3. Regression results for model excluding usual gait speed.

Predictors	B	SE	β	*p*-Value	VIF
Age (year)	0.0006	0.1328	0.0498	0.7087	1.45
Sex (1 = female, 0 = male)	0.0055	0.2442	0.0382	0.8761	4.89
Height (cm)	−0.0016	0.2538	−0.1971	0.4406	5.28
Weight (kg)	0.0013	0.3249	0.188	0.565	8.66
SMM (kg)	0.0006	0.2401	0.0463	0.8477	4.73
Fat (%)	0.0015	0.2392	0.167	0.4877	4.69
SPPB	−0.0033	0.1318	−0.0587	0.6575	1.42
30 s STS counts	−0.0006	0.1235	−0.0535	0.6668	1.25
Daily mean steps	0.0000006	0.1204	0.2895	0.0194 *	1.19
RPE	0.0003	0.1472	0.0112	0.9396	1.78
Subjective health status	0.0405	0.1393	0.2857	0.0446 *	1.59
R^2	0.281				
F for Model	2.09 *				

* $p < 0.05$.

Table S4. Regression results for model each digital lifelogs individually.

Added Variable	R^2	F	*p*	B	SE	β	*p*-Value	VIF
Usual Gait Speed	0.2234	2.23	0.037 *	−0.1431	0.1193	−0.2981	0.0151 *	1.14
30 s STS counts	0.1473	1.339	0.242	−0.0006	0.1305	−0.0514	0.6952	1.24
Daily mean steps	0.2077	2.032	0.057	0.000005	0.1157	0.2559	0.0307 *	1.05
RPE	0.1834	1.74	0.107	0.0063	0.1287	0.2192	0.0935	1.26
Subjective health status	0.1974	1.906	0.075	0.0349	0.1225	0.2461	0.049 *	1.16

* $p < 0.05$.

Table S5. Robust regression (Huber's T) results.

Predictors	B	SE	β	*p*-Value	VIF
Age (year)	−0.0003	−0.0222	0.1219	0.8553	1.48
Sex (1 = female, 0 = male)	0.0104	0.0721	0.2216	0.7450	4.89
Height (cm)	−0.0018	−0.2322	0.2305	0.3137	5.3
Weight (kg)	0.0011	0.1574	0.2968	0.5957	8.78
SMM (kg)	0.0008	0.0684	0.2187	0.7545	4.77
Fat (%)	0.0005	0.0588	0.2181	0.7875	4.74
SPPB	−0.0042	−0.0751	0.1205	0.5332	1.45
Usual gait speed (m/s)	−0.0962	−0.2004	0.1113	0.0716	1.23
30 s STS counts	−0.0006	−0.0490	0.1132	0.6652	1.28
Daily mean steps	0.000005	0.2490	0.1096	0.0230 *	1.2
RPE	−0.0011	−0.0369	0.1346	0.7841	1.81
Subjective health status	0.0288	0.2029	0.1271	0.1105	1.61
R^2	0.3200				
χ^2	26.667 *				

* $p < 0.05$.
